# Research on anti-swing control system of slewing crane based on fuzzy PID

**DOI:** 10.1371/journal.pone.0311701

**Published:** 2024-10-25

**Authors:** Manman Xu, Lei Liu, Jiajun Wang, Yonghong Wu, Xiangdong Wang, Yongming Liu

**Affiliations:** 1 Anhui Polytechnic University, Wuhu, China; 2 Anhui Key Laboratory of Mine Intelligent Equipment and Technology, Anhui University of Science & Technology, Huainan, China; 3 Wuhu Magnetic Wheel Transmission Technology Co., LTD, Wuhu, China; Imperial College London, UNITED KINGDOM OF GREAT BRITAIN AND NORTHERN IRELAND

## Abstract

**Background:**

The slewing crane is easily affected by the wind and the manipulation level of the operator when it is working, which in turn impacts its swing angle, affects the working efficiency and safety of the crane.

**Objective:**

Toimprove the operation safety andreduce the swing angle, this study design and research the anti-swing control system for the slewing crane.

**Methods:**

Firstly, based on the Lagrange equation, the dynamic model of the crane hoisting system is established. Then, the mechanical anti-swing mechanism driven by a four-motor rope is established. Finally, based on fuzzy PID control, an anti-swing platform is established, and the performance of anti-swing control is verified.

**Results:**

Compared to no anti-swing control, the in-plane swing angle θ_1_ and out-of-plane swing angle θ_2_ of the lifting arm are reduced by 88% and 75% respectively.

**Conclusion:**

The results show that the four-motor rope-driven anti-swing mechanism can achieve better anti-swing effect, which provides an effective swing suppression method for the slewing crane in the actual operation.

## 1 Introduction

As an underactuated mechanism, the crane system exhibits a swinging trend due to its lack of flexibility, insufficient constraint, and external vibration during normal operations [1]. It not only affects the accuracy of hoisting goods, but also seriously affects production efficiency and increase security risks.

Thomas Gustafsson et al. investigated the feedback control problem and pitching motion of the slewing crane by establishing a dynamic model and designing a feedback controller, and successfully realized the swing suppression in the experiment [2].

Ho-Hoon Lee and Sung-Kun Cho proposed an anti-swing control system that integrates position servo control and fuzzy logic control. It controls the crane position and the rope length, while also reducing the oscillation of the lifting weight. Its anti-swing control effect has been verified by experiments [3].

Kunihiko Nakazono et al. combined genetic algorithm with BP neural network control to effectively suppress swing by adjusting rotational speed. It is suitable for anti-swing control under uncertain wind conditions [4,5]. Through dynamic analysis and research of the crane, Yuan Zhigang and Zang Tiegang designed a time-delay positioning feedback control system to reduce the swing [6,7]. Zhao Minghui established the mathematical model of the bridge crane, and proposed a fuzzy control anti-pendulum method based on genetic algorithm. Simulation analysis shows that this method is effective for crane anti-swing [8].

Kang Xinyu et al. designed an improved Active Disturbance Rejection Controller to solve the tower crane more underactuated and nonlinear. They proposed a smooth and non-linear function to reduce the high-frequency oscillation of the system at steady-state. And it constructed a new type of Extended State Observer to improve the dynamic response performance of the system. Numerical simulation results showed that the proposed controller has superior control performance and strong robustness [9].

Xuan H L et al. presented an adaptive backstepping hierarchical sliding mode control algorithm for uncertain 3D overhead crane model. It used RBF neural network to approximate nonlinear function of crane, design hierarchical sliding mode controller based on Lyapunov theory [10].

Guo Q et al. proposed a coordinated control method for the track and trolley of the double pendulum crane to improve the working efficiency of the crane, which realizes the anti-swing control of the double pendulum crane in three-dimensional movement mode [11].

Tingqi Zhao et al. proposed a novel fuzzy sliding mode anti-sway controller by combining the in-plane angle and the out-plane angle with the fuzzy sliding mode technology, with the aim of addressing the existing problems facing simple control algorithms, low control accuracy of ship-mounted crane by the mechanical anti-sway method [12].

In summary, the research subjects are generally simplified, combining theoretical analysis with empirical testing, using different methods to control the crane movement or design different anti-swing structures. It is worth noting that in most studies, control methods or structures are complex, difficult to implement, and difficult to generalize. The existing rope-driven anti-swing devices mostly belong to spatial flexible cable mechanisms, and their installation requires dismantling the original structure, which limits the operating range [13].

Lu Biao et al. proposed a high-performance nonlinear controller, which can better suppress the payload swing due to the incorporation of more swing-related information. The method can well conquer the payload mass uncertainty with an adaption law, so that many corresponding adverse effects are eliminated [14].

Sun N, et al. provided explicit Lyapunov-based analysis to rigorously prove that the equilibrium point of the closed-loop system is almost globally asymptotically stable, without any approximation to the original nonlinear dynamics. The first closed-loop control method can achieve control for an underactuated double-pendulum crane with merely OFB and theoretically-guaranteed saturated control efforts [15].

Frikha S, et al. drawn out the equivalent controller and the switching controller for gantry crane, on the base of the sliding mode control principle. The adaptive control laws for these controllers were deduced from Lyapunov’s stable criteria to asymptotically stabilize the sliding surfaces [16].

Aiming at these challenges, an anti-swing system based on fuzzy PID with simple structure and reliable operation is designed, and a fuzzy PID real-time control device is realized. This system improves the efficiency of the control system, enhances the anti-swing effect, and emphasizes the test repeatability.

## 2 Materials and methods

### 2.1 Dynamics modeling and analysis of the crane hoisting system

**Fig 1:**
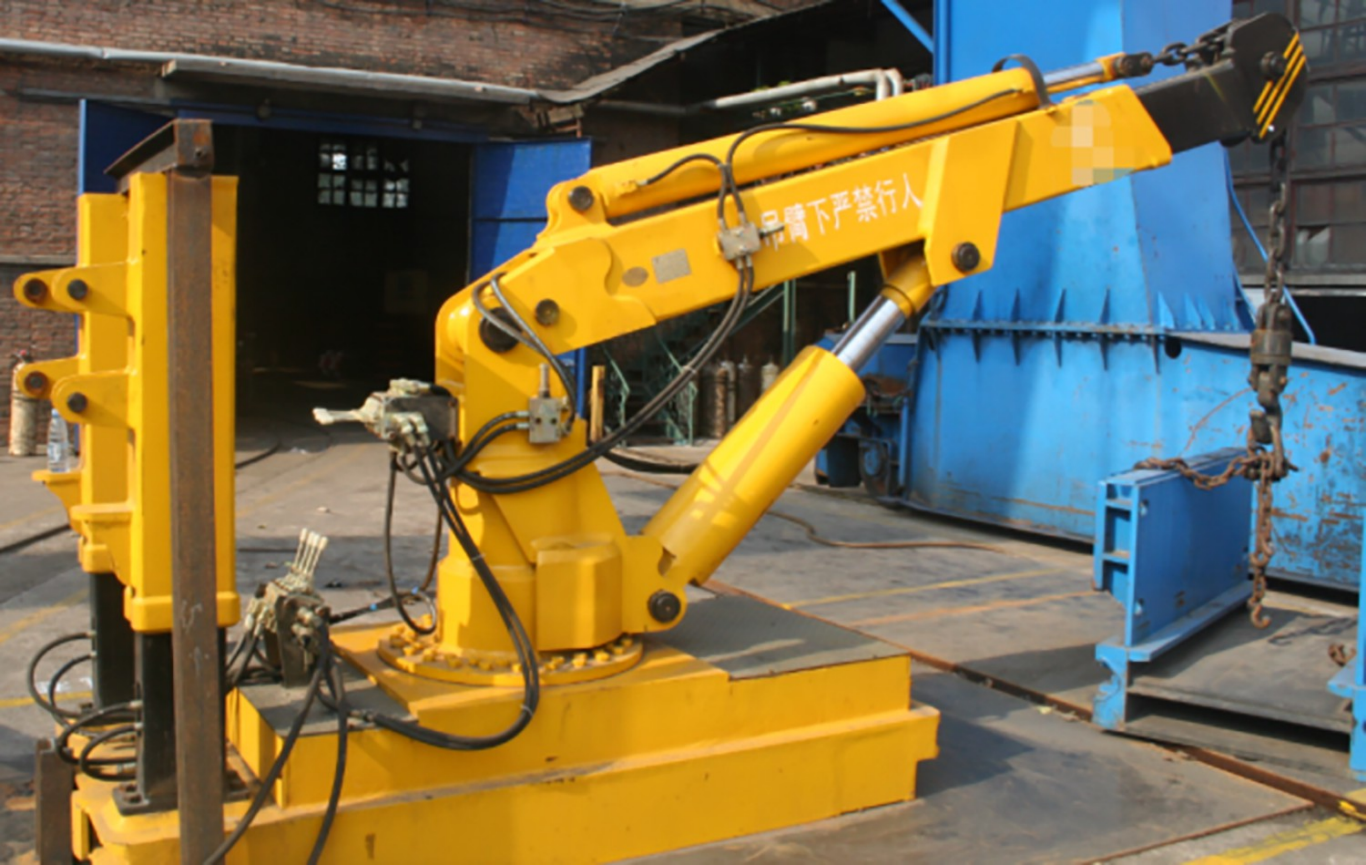
Slewing crane physical model.

The slewing crane in this study is mainly used for the installation and disassembly of heavy equipment under the mine. The total weight of the crane is 9t and the capacity weight can reach 5t. Fig 1 is the slewing crane physical model, and shows the physical structure of the crane.

**Fig 2:**
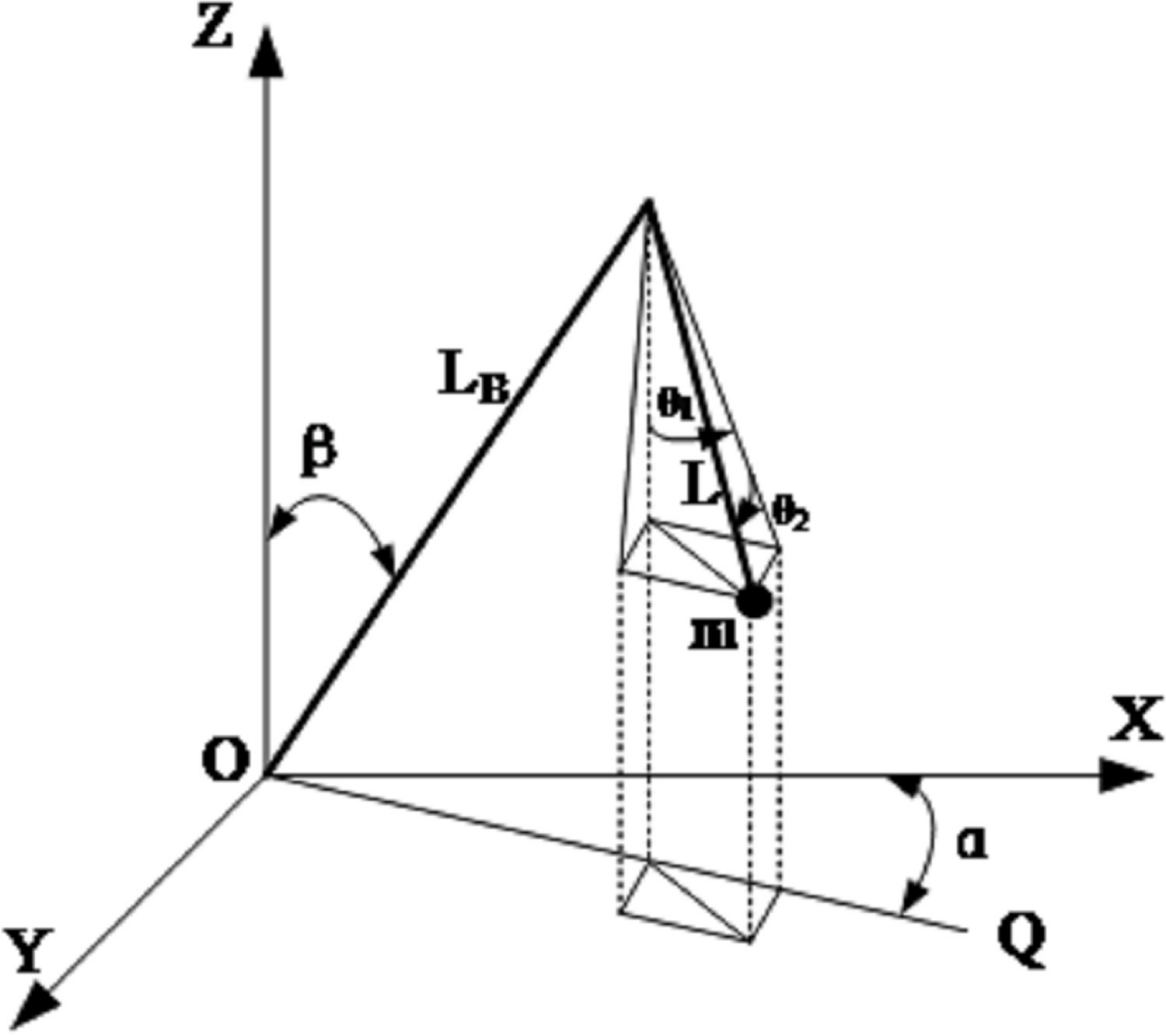
Physical model of the slewing crane.

The crane boom adopts a telescopic box type straight arm structure. Among them, the telescopic sleeve relates to the turntable, which is convenient for the jib to rotate on the pitching plane. Moreover, the bottom of the telescopic sleeve is hinged with the piston rod of the variable amplitude hydraulic cylinder, and the cylinder body of the variable amplitude hydraulic cylinder is hinged with the turntable, so that the variable amplitude hydraulic cylinder drives the boom to rotate in the variable amplitude plane. In addition, a hydraulic cylinder is installed between the telescopic sleeve and the two telescopic arms to ensure that the telescopic arms can be extended horizontally. The crane boom adopts the sequential expansion mode [17–21]. Fig 2 is the physical model of the slewing crane. And its parameters are shown in Table 1, respectively.

**Table 1:** Meaning of the symbols.

Symbol	Meaning
*θ1(rad)*	Inside angle of ZOQ plane
*θ2(rad)*	Outside angle of ZOQ plane
*α(rad)*	Rotation angle
*β(rad)*	Pitch angle
*L(m)*	Rope length
*LB(m)*	Hanging arm length
*m(kg)*	Hoisting weight

In this model, the swing of a hanging object in space is measured with the inside angle of ZOQ plane of θ_1_ and the outside angle of ZOQ plane of θ_2_. The rope length is L, the hoisting weight is m, and the hanging arm length is L_B_. The angle between the arm plane and the X-axis is the rotation angle α, and the residual angle of the crane boom arm and the Z-axis is the pitch angle β, so the position of the load center in the inertial coordinate system is:

$$\left\{ \begin{array}{l} x_{load}=L_{B}\cos\alpha \sin\beta +L\cos\alpha \sin\theta_{1}\cos\theta_{2}-L\sin\alpha \sin\theta_{2}\\ y_{load}=L_{B}\sin\alpha \sin\beta +L\sin\alpha \sin\theta_{1}\cos\theta_{2}+L\cos\alpha \sin\theta_{2}\\ z_{load}=L_{B}\cos\beta -L\cos\theta_{1}\cos\theta_{2} \end{array}\right.$$ (1)

In Eq (1), α, β, θ_1_, θ_2_ and L all change with time. By further derivation, the kinetic energy of the lifting swing can be calculated by Eq (2).


$$T=\frac{1}{2}m({\dot{x}}_{load}^{2}+{\dot{y}}_{load}^{2}+{\dot{z}}_{load}^{2})$$ (2)

Taking the zero potential energy point of the plane where the rotating base of the slewing crane is located, then the potential energy of the crane is as follows.


$$V=mgz_{load}=mg(L_{B}\cos\beta -L\cos\theta_{1}\cos\theta_{2})$$ (3)

Considering the above formulas, the Lagrange equation in the system is:

L=T−V (4)

Aiming at the anti-swing control of the crane hoisting system, the swing angles θ_1_ and θ_2_ are selected as the generalized coordinate system, and according to the Lagrange equation:

$$\{\begin{array}{c} \frac{d}{dt}(\frac{\partial L}{\partial \dot{\theta_{1}}})-\frac{\partial L}{\partial \theta_{1}}=Q_{\theta_{1}}\\ \frac{d}{dt}(\frac{\partial L}{\partial \dot{\theta_{2}}})-\frac{\partial L}{\partial \theta_{2}}=Q_{\theta_{2}} \end{array}$$ (5)

In Eq (5), Q_θ1_ and Q_θ2_ are the generalized forces subjected to the hoisting weight. In practical operation, friction is inevitable between the rope and the pulley of the crane lifting heavy objects; In addition, the hoisting weight of the crane is also affected by air resistance and friction resistance. Due to the randomness of this part, it is approximately zero in the research process.

In addition, when the swing angle is small, certain items in the formula can be approximately calculated during analysis. Considering the random disturbance that affects the swing of the lifting rope, the dynamic equation of the lifting system is as follows:

$$\left\{ \begin{array}{l} {\ddot{\theta }}_{1}=({\dot{\alpha }}^{2}+\frac{L_{B}\dot{\beta }cos\beta }{L}+\frac{L_{B}\dot{\beta }sin\beta }{L}-\frac{g}{L})\theta_{1}-\frac{2L}{L}{\dot{\theta }}_{1}+\dot{\alpha }{\dot{\beta }}_{2}\\ +\ddot{\alpha }\beta_{2}+\frac{L_{B}{\dot{\beta }}^{2}sin\beta }{L}+\frac{L_{B}{\dot{\alpha }}^{2}sin\beta }{L}-\frac{L_{B}{\ddot{\beta }}^{2}cos\beta }{L}-\mu_{1}\dot{\theta_{1}}\\ {\ddot{\theta }}_{2}=({\dot{\alpha }}^{2}+\frac{L_{B}\dot{\beta }cos\beta }{L}+\frac{L_{B}\dot{\beta }sin\beta }{L}-\frac{g}{L})\theta_{2}-\frac{2L}{L}{\dot{\theta }}_{2}-\dot{\alpha }{\dot{\beta }}_{1}\\ -\ddot{\alpha }\beta_{1}-\frac{L_{B}{\ddot{\alpha }}^{2}sin\beta }{L}-\frac{2L_{B}\dot{\alpha }\dot{\beta }cos\beta }{L}-\mu_{2}\dot{\theta_{2}} \end{array}\right.$$ (6)

To sum up, the lifting load effect of crane is the comprehensive effect of the nonlinear combination of boom rotation and pitching motion, which couples the swing of heavy objects with movement of crane. The swing of the lifting weight is related to the pitch angle, turn angle and rope length.

**Fig 3:**
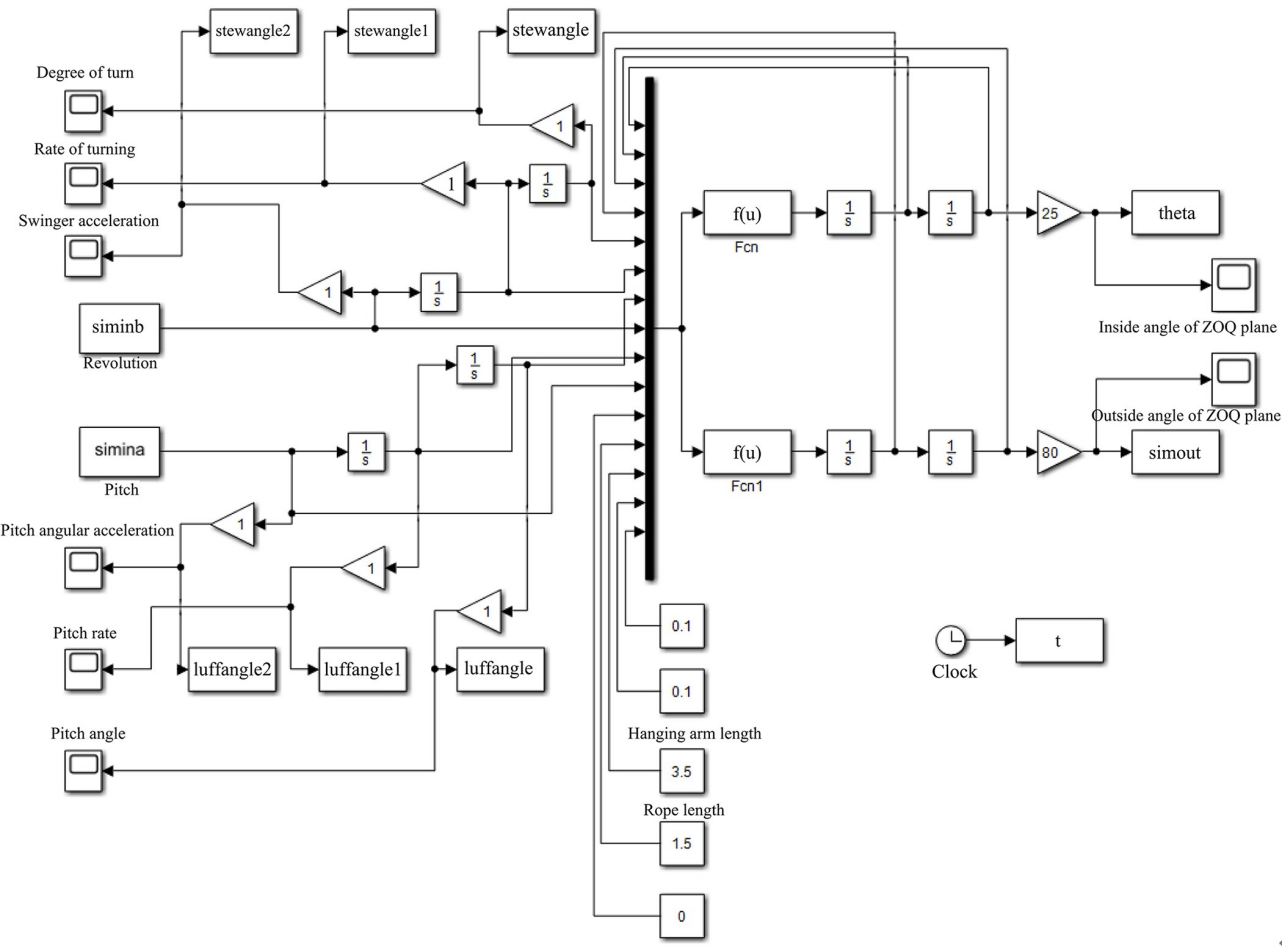
Numerical simulation model of the slewing crane.

According to Eq (6), crane rotation angle α, pitch angle β, and rope length L are selected as system inputs. In the simulation process, according to the actual working conditions, the crane arm length L_B_ is 3.5m. The numerical simulation model of rotary crane was established, as shown in Fig 3. On the left side of the model is input angle data. The input data is converted to the formulas Fcn and Fcn1 for calculation. Finally, the results are obtained and the outputs θ_1_ and θ_2_ on the right are compared with θ_1_ and θ_2_ in Fig 3 to illustrate the interaction between the input angle and the output values θ_1_ and θ_2_.

### 2.2 Anti-swing control system design

A four-motor rope-driven mechanical anti-swing mechanism based on fuzzy PID and a closed-loop control system based on dSPACE are designed. The basic principle is that the computer measures the swing angle of the crane, determines the closed-loop control strategy, and then activates the motor control system through the lower computer to mitigate the swing.

**Fig 4:**
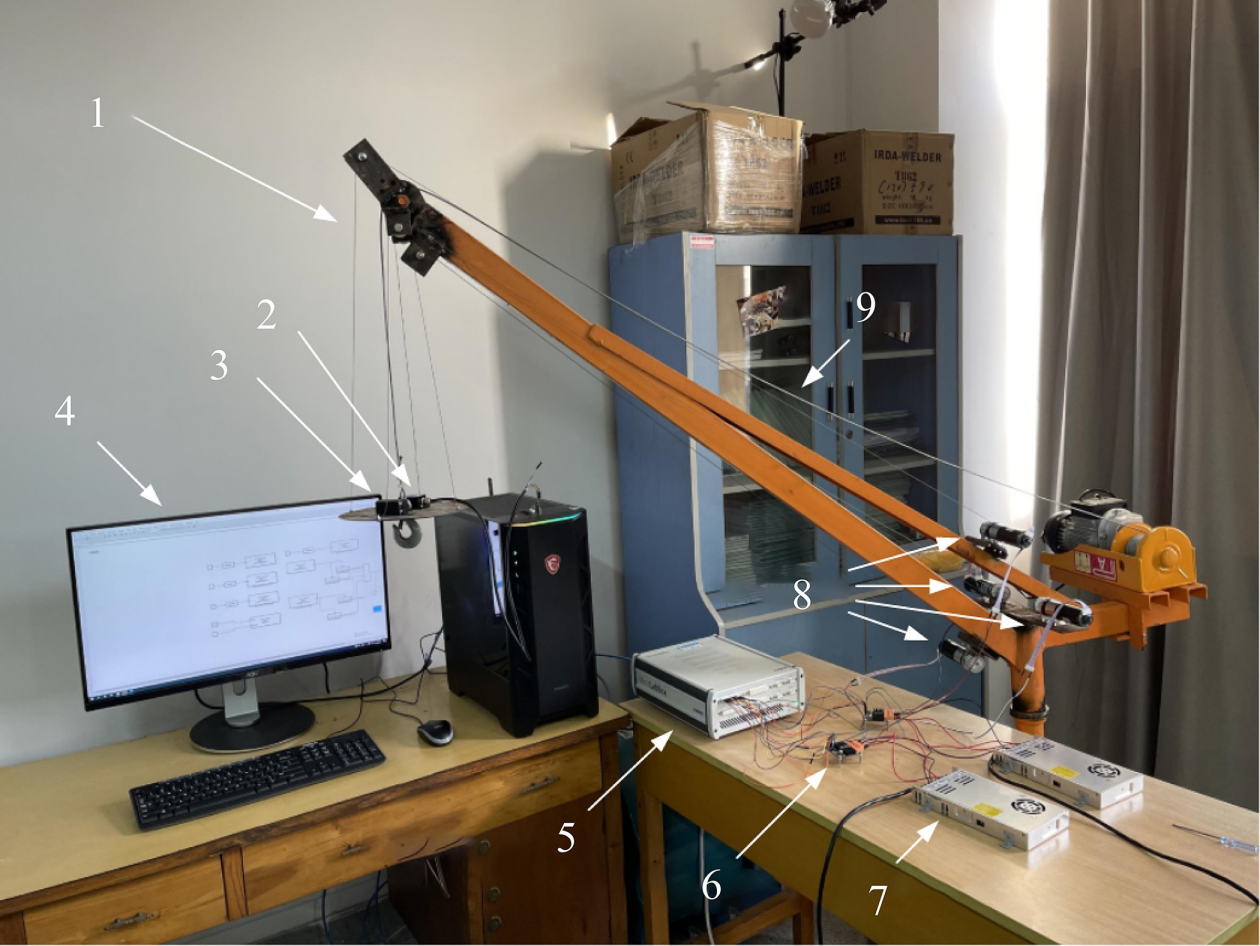
Anti-swing structure physical diagram of crane rope drive system. 1-anti-swing arm; 2-inclination sensor; 3- traction rope; 4-computer; 5-dSPACE MicroLabBox DS1202; 6-motor driver; 7-switching power supply; 8-anti-swing motor; 9- main lifting rope.

The anti-swing structure physical diagram of crane rope drive systemis shown in Fig 4. The structure comprises four anti-swing arms arranged symmetrically on the boom. A small pulley with guide rope is installed on the anti-swing arm, and four traction ropes are driven by the anti-swing motor to inhibit the swing of the load. The main lifting rope of the small crane is connected to the center of the lifting plate through the main pulley on the top of the boom, and the other end is fixed on the storage rope device of the main motor. In the process of rotating movement, when the lifting weight swings in a certain direction, the traction rope will drive the lifting disc to move in the opposite direction, inhibit the swing of the lifting weight, so that the lifting system is in a stable state, thus stabilizing the load of the crane.

To measure the tilt angle of the crane weight, SIN-VT series sensors are selected, and the product performance parameters are shown in Table 2. The selected inclination sensor detects the acceleration, angular velocity and angle of the three axes (X, Y and Z axes). The swing angle of the tilt sensor rotating around the Y-axis is θ_2_, and the swing angle of the tilt sensor rotating around the X-axis is θ_1_.

**Table 2:** Performance parameters of the SIN-VT inclination sensor.

Product Model	SIN-VT
Operating temperature requirements	-40°C ~ 85°C
Range	0°~±90°
Full range accuracy	0.1°
Response time	0.01 s
Anti-vibration	10 grms; 10~1000 Hz

### 2.3 Selection of anti-swing motor

The four anti-swing motors in this experiment are MD36NP71_24V DC motors. The parameters of the DC motor are as follows: the motor resistance is 10.43 Ω, the motor inductance is 4.4e-3 H, the torque constant is 0.05 N·m/A, the proportional coefficient is 0.05 V·s/rad, the motor inertia is 1e-5 kg·m2, the viscous motor damping is 1e-6 N·s/rad, the reduction ratio of anti-swing motor is 71:1, and the working efficiency of the planetary gear reducer is about 0.75.

To achieve the precise control of the swing amplitude, it is necessary to model and simulate the anti-swing DC motor [22]. Among them, the DC motor has three important characteristics:

Electromagnetic torque Te is calculated as follows, where Kt is the torque constant and ia is the current of the armature.


$$T_{e}=K_{t}\cdot i_{a}$$ (7)

The back electromotive force e generated by the DC motor is proportional to the rotational angular velocity ω. The scale factor is Ke.


$$e=K_{e}\cdot \omega =K_{e}\cdot \frac{d\theta }{dt}$$ (8)

When the DC motor is running without load, the input voltage is equal to the back electromotive force. So that the Kt and Ke are the same quantity electrically.


$$K_{t}=K_{e}$$ (9)

In summary, the balance relationship between the electromagnetic torque Te generated by the DC motor and the torque T transmitted to the load is as follows:

$$T_{e}=T+(J\overset{¨}{\theta }+B_{m}\dot{\theta })$$ (10)

Where, θ, J and Bm respectively represent the rotation Angle, the moment of inertia, and the viscous friction force during rotation. Kirchhoff’s voltage law gives the following formula.


$$v=R_{a}\cdot i_{a}+L_{a}\cdot \frac{di_{a}}{dt}+K_{e}\cdot \frac{d\theta }{dt}$$ (11)

Where, v is the input voltage; R represents the rotor internal resistance; ia and La rotor current and rotor inductance, respectively. After the Laplace conversion is available:

$$v(s)=R_{a}\cdot i_{a}(s)+L_{a}\cdot s\cdot i_{a}(s)+K_{e}\cdot s\cdot \theta (s)$$ (12)

Upon conclusion of these derivations, the DC motor model is formulated as follows:

$$i_{a}(s)=\frac{1}{L_{a}\cdot s+R_{a}}v(s)-\frac{K_{e}\cdot s}{L_{a}\cdot s+R_{a}}\theta (s)$$ (13)

Accordingly, the motor model is established in MATLAB/Simulink based on Eq 13.

**Fig 5:**
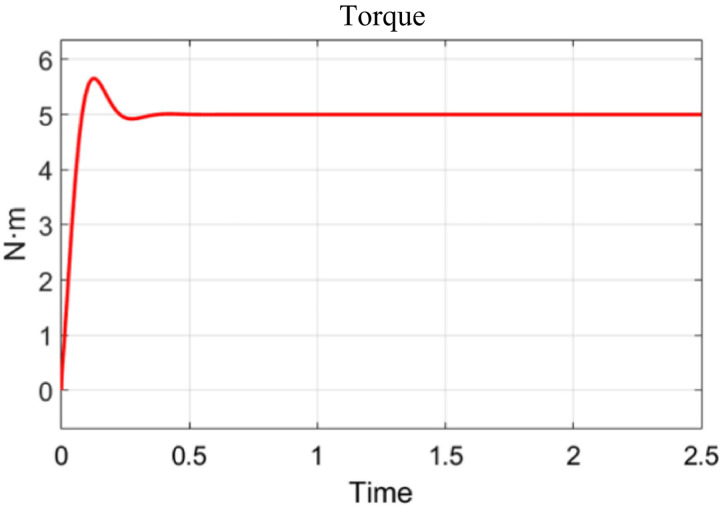
Schematic diagram of the torque output.

Since DC motors have certain limits on the input voltage, the motor may be damaged when the controller’s input value exceeds the motor’s safety threshold. Therefore, the upper limit of the motor safety voltage is the upper limit of the controller regulation. Fig 5 is the schematic diagram of the torque output. The DC motor model is run in Simulink, and the results are shown in Fig 5. Its output torque is 5N·m, far higher than the target, and the response time can also meet the requirements.

**Fig 6:**
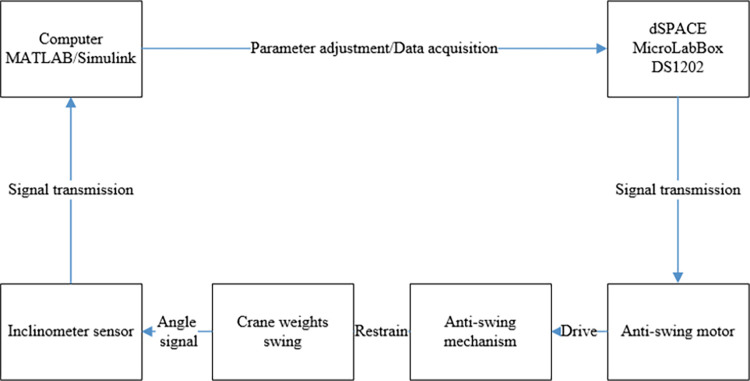
Flow of pendulum control system.

The dSPACE MicroLabBox semi-physical simulation platform was used to design the anti-swing mechanism control system of rope drive system [23–30]. After the simulation platform transmits the signal to the anti-swing motor, the tilt sensor receives the swing signal of the crane, and then transmits it to the computer and the simulation platform for parameter adjustment, thereby achieving effective suppression of the swing of the lifting weight. Fig 6 is the Flow of pendulum control system. The flow chart of the anti-swing control system is shown in Fig 6.

Based on the design of the anti-swing mechanism control system, the PID control method of the DC motor is further explored to improve the anti-swing effect of the system [31–34]. According to the working principle of PID, the actuator is defined to complete the anti-swing action, and the variables to be controlled are determined [35–37]. The PID speed control module of the anti-swing motor is established in the computer program. Simultaneously, adjusting the parameters of the PID controller to achieve the best performance of the motor.

Based on the advantages of small overshoot, fast response and strong robustness of fuzzy PID controller, the simulation model of anti-swing motor control is established by MATLAB/Simulink [38].

Calculating the deviation between the current speed and the set speed of the motor, the change of the current deviation and the last deviation, setting parameters in MATLAB/Simulink, and perform fuzzy PID control for the speed of a single anti-pendulum direct current motor.

**Fig 7:**
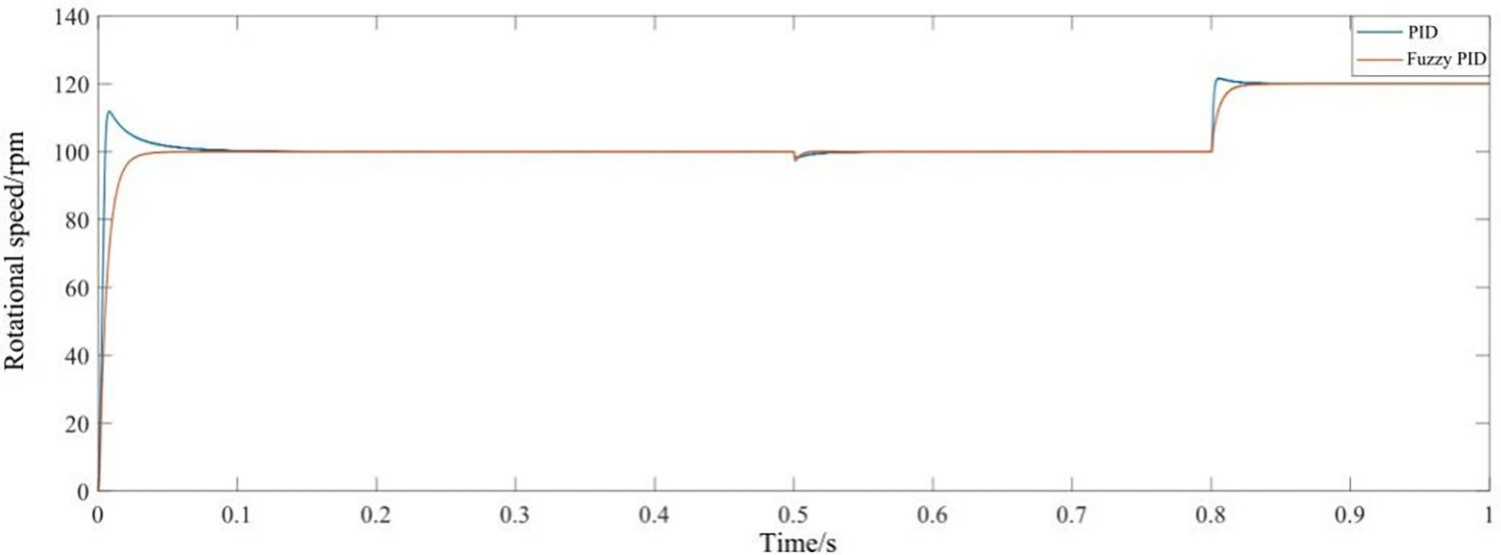
Comparison between the fuzzy PID control and the traditional PID control.

The relative error is set to 1e-6, the simulation time is 1s, the given speed is 100 rpm, and the motor starts with no load. At 0.5 s, heavy lifting load is added, and the speed is increased to 120rpm at 0.8 s. Fig 7 shows the comparison between the fuzzy PID control and the traditional PID control. Compared with traditional PID control, fuzzy PID controller has the advantages of small overshoot, fast response and strong robustness in Table 3.

**Table 3:** Comparison of the rotational speed of the different controllers.

Speed Controller		Fuzzy PID	PID
Start	Overshoot	Not	13%
	Response time(s)	0.04	0.12
Applied Load	Decrease amount	2.8%	1.8%
	Adjust the time(s)	0.012	0.06
Increase Speed	Overshoot	Not	1.7%
	Adjust the time(s)	0.04	0.06

## 3 Results and discussion

Based on the established anti-swing experiment platform and fuzzy PID anti-swing control method, the dSPACE Micro-LabBox semi-physical simulation system is used to control the DC motor in the anti-swing structure, and relevant experiments are carried out to verify the actual effect of the anti-swing mechanism.

**Fig 8:**
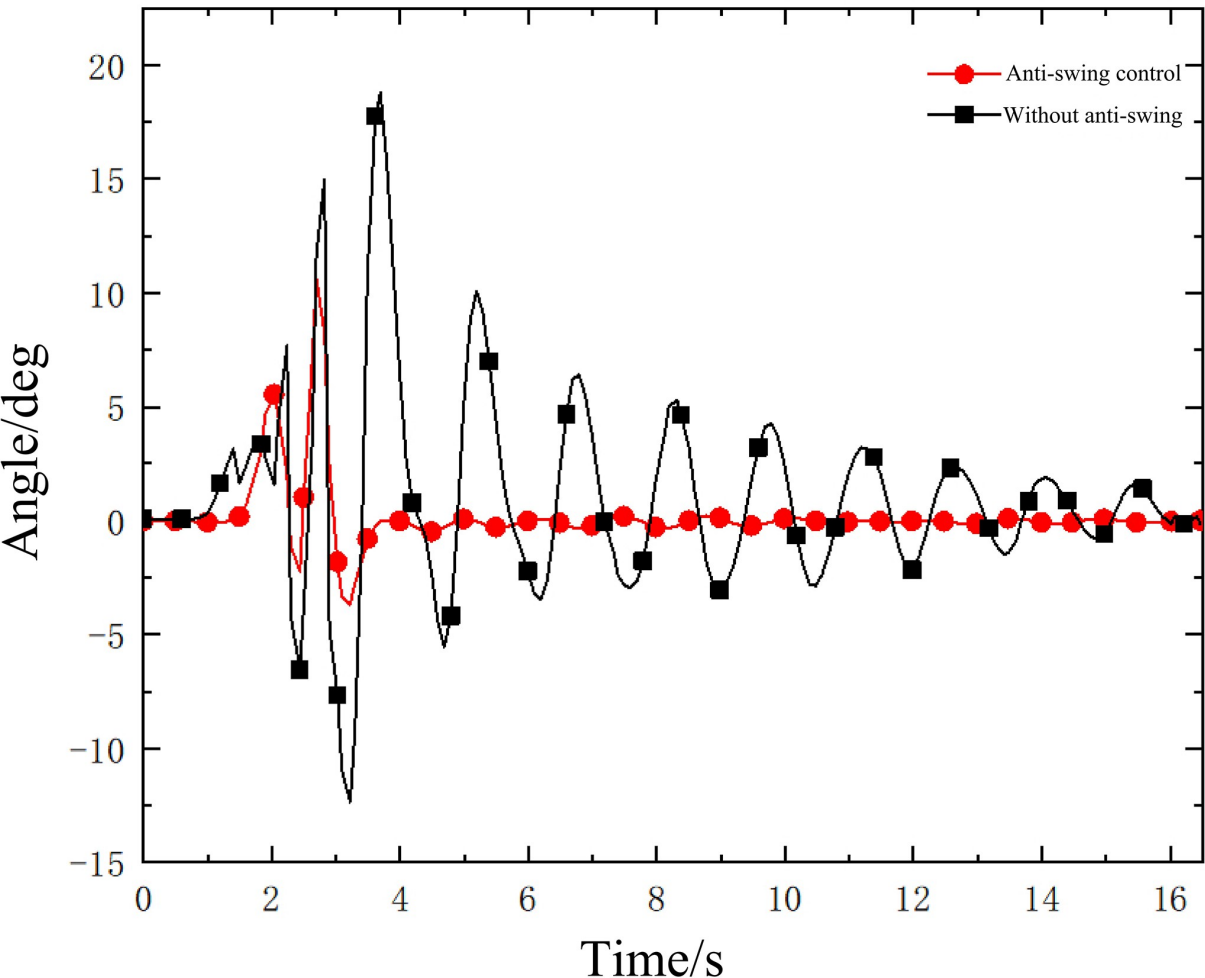
Anti-swing angle θ_1_ of hanging weight.

**Fig 9:**
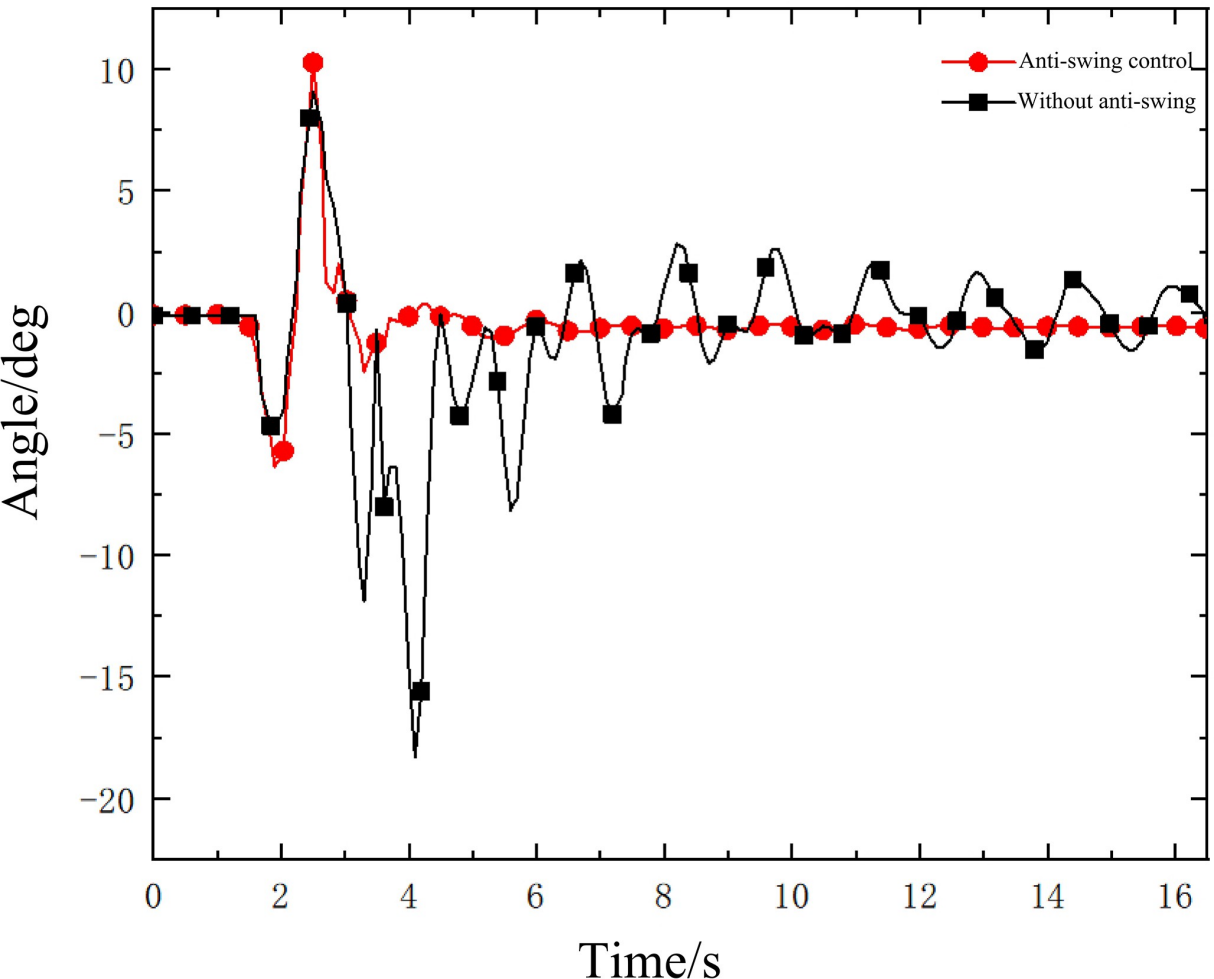
Anti-swing angle θ_2_ of hanging weight.

The swing angle of the lifting load inside and outside the boom plane is shown in Figs 8 and 9. Fig 8 is the anti-swing angle θ_1_ of hanging weight and Fig 9 is the anti-swing angle θ_2_ of hanging weight. It can be seen from the hoisting swing angle monitored by the inclination sensor that under the action of the anti-swing control system, the swing of the rope hoisting system of the small crane has been effectively suppressed. At the beginning of the experiment, the maximum value of angle θ_1_ is 6° and the maximum value of angle θ_2_ is 8°. After 5 s of the experiment, the swing of the lifting object decreases rapidly to a stable state. The angle θ_1_ is 0.7° and the angle θ_2_ is 2°. The oscillation degree of the lifting system is not large. Among them, the inhibition rate of the in-plane swing angle reached 88%, and the inhibition rate of the out-of-plane swing angle reached 75%. By comparing the swing angle of hoisting weight under anti-swing control and without anti-swing conditions, the effectiveness of the mechanical anti-swing mechanism and its anti-swing control system based on four-motor rope drive is further verified intuitively. This experiment provides a feasible solution for suppressing load swing.

## 4 Conclusions

This study focuses on the swing problem of the traditional slewing crane hoisting system. Based on the Lagrange equation, the dynamic model of the crane hoisting system is established. The mechanical anti-swing mechanism driven by a four-motor rope is established. A mechanical anti-swing mechanism based on four-motor rope drive by analyzing its dynamic characteristics, establishing a dynamic model, and conducting simulation experiments. Based on fuzzy PID control, an anti-swing platform is established, and the performance of anti-swing control is verified. The fuzzy PID control is applied to the anti-swing structure of the DC motor, and a comprehensive anti-swing control system is formed. The main conclusions are as follows:

(1) Based on the existing research conclusions domestically and internationally, through the analysis of the swing characteristics of the crane rope lifting system, the dynamic model of the crane hoisting system is established based on the Lagrange equation, the study proposes a straightforward mechanical anti-swing structure based on fuzzy PID and a closed-loop control system based on dSPACE using the four-motor rope-driven.

(2) Firstly, the Lagrange function is used to calculate the in-plane angle and the anti-swing angle, and the numerical simulation model is established to obtain the transformation images of θ_1_ and θ_2_. Thereafter, the control strategy of the anti-swing control system and anti-swing motor control system is designed by dSPACE MicroLabBox hardware-in-the-loop simulation platform, and the structure is verified by experiments. Compared with the case without anti-swing control, θ_1_ and θ_2_ decreased by 88% and 75%, respectively. These results unequivocally affirm the efficacy of the proposed configuration in mitigating crane anti-swing phenomenon.
